# Parity and thyroid cancer risk: a meta‐analysis of epidemiological studies

**DOI:** 10.1002/cam4.604

**Published:** 2015-12-29

**Authors:** Jingjing Zhu, Xiao Zhu, Chao Tu, Yuan‐Yuan Li, Ke‐Qing Qian, Cheng Jiang, Tong‐Bao Feng, Changwei Li, Guang Jian Liu, Lang Wu

**Affiliations:** ^1^ Division of EpidemiologyDepartment of MedicineVanderbilt Epidemiology CenterVanderbilt‐Ingram Cancer CenterVanderbilt University School of MedicineNashvilleTennessee37203; ^2^ Program of Quantitative Methods in EducationUniversity of MinnesotaMinneapolisMinnesota55455; ^3^ Guangdong Provincial Key Laboratory of Medical Molecular DiagnosticsDongguan Scientific Research CenterGuangdong Medical UniversityDongguan523808China; ^4^ Oncology Institutethe Affiliated Hospital of Nanjing Medical UniversityChangzhou No.2 People's HospitalChangzhouJiangsu213003China; ^5^ Department of Hematologythe Affiliated Hospital of Xuzhou Medical CollegeXuzhouJiangsu221000China; ^6^ Department of Neurologythe Affiliated Brain Hospital of Nanjing Medical UniversityNanjingJiangsu210000China; ^7^ Department of EpidemiologyTulane University School of Public Health and Tropical MedicineNew OrleansLouisiana70112; ^8^ Department of NeurologyTaihe Hospital Affiliated to Hubei University of MedicineShiyanHubei442000China

**Keywords:** Epidemiology, meta‐analysis, parity, risk, thyroid cancer

## Abstract

Although observational studies have assessed the relationship between parity and thyroid cancer risk, the findings are inconsistent. To quantitatively assess the association, we conducted a systematic review and meta‐analysis. PubMed and Embase were searched up to January 2015. Prospective or case–control studies that evaluated the association between parity and thyroid cancer risk were included. We used the fixed‐effects model to pool risk estimates. After literature search, 10 prospective studies, 12 case‐control studies and 1 pooled analysis of 14 case‐control studies including 8860 patients were identified. The studies had fair methodological quality. Pooled analysis suggested that there was a significant association between parity and risk of thyroid cancer (RR for parous versus nulliparous: 1.09, 95% CI 1.03‐1.15; I2=33.4%). The positive association persisted in almost all strata of subgroup analyses based on study design, location, study quality, type of controls, and confounder adjustment, although in some strata statistical significance was not detected. By evaluating the number of parity, we identified that both parity number of 2 versus nulliparous and parity number of 3 versus nulliparous demonstrated significant positive associations (RR=1.11, 95% CI 1.01‐1.22; I2=31.1% and RR=1.16, 95% CI 1.01‐1.33; I2=19.6% respectively). The dose‐response analysis suggested neither a non‐linear nor linear relationship between the number of parity and thyroid cancer risk. In conclusion, this meta‐analysis suggests a potential association between parity and risk of thyroid cancer in females. However, the lack of detection of a dose‐response relationship suggests that further studies are needed to better understand the relationship.

## Introduction

As the most common type of the endocrine malignancies, thyroid cancer causes a large number of deaths that is higher than the combined number of all other endocrine cancers 1. It is estimated that in the US, 15,220 males and 47,230 females will newly develop thyroid cancer in 2015 2. A large proportion of etiology for certain subtypes of thyroid cancer, such as medullary thyroid cancer and familial papillary thyroid cancer, can be attributed to genetic factors 3, 4. Research also has demonstrated that exposure to ionizing radiation, iodine availability, body mass index (BMI), height, vegetable consumption, smoking, alcohol drinking, diabetes, and obesity can influence individual's risk of developing thyroid cancer 5, 6, 7, 8, 9, 10, 11. However, to date, a large proportion of the etiology of thyroid cancer has not been fully understood. Considering a huge difference in incidence of thyroid cancer between males and females, it may be warranted to hypothesize that reproductive factors may play roles in the etiology. This hypothesis is also aligned with the fact that the incidence rate of thyroid cancer in females is highest during the reproductive years 12. Oral contraceptives (OC) use has been suggested to be associated with thyroid cancer risk in a dose–response relationship, based on evidence from prospective studies 13. As another representative reproductive factor, parity is also hypothesized to be associated with thyroid cancer risk. To date, numerous studies have investigated the association between parity and risk of thyroid cancer, but yielded inconsistent findings. It was demonstrated that ever giving birth to children conferred a higher risk of developing thyroid cancer in women by Mctiernan et al. 14. Several other studies also supported that a higher number of parity was associated with increased risk 15, 16. However, a study conducted in Japan supported an inverse conclusion 17 and many other studies revealed nonsignificant associations 12, 18, 19, 20, 21, 22. We thus conducted this systematic review and meta‐analysis for summarizing available evidence from epidemiological studies to assess the association between parity and thyroid cancer risk in females, including evaluating the dose–response relationship.

## Methods

This meta‐analysis was performed in accordance with the MOOSE guideline 23.

### Data sources and search strategies

A search of PubMed (MEDLINE) and Embase was conducted from each database's inception to January 2015 for studies of humans published in English. We used the following search keywords and Medical Subject Heading terms: (parity OR pregnancy OR livebirth OR reproductive OR reproduction OR reproductive factors) AND (papillary OR follicular OR thyroid) AND (cancer OR neoplasm OR carcinoma OR tumor OR adenoma OR cancers OR neoplasms OR carcinomas OR tumors OR adenomas). We also reviewed references of relevant review articles to identify additional potential studies.

### Study selection

Studies were eligible if they (1) were case–control studies or prospective studies; (2) evaluated the association between parity and risk of thyroid cancer; (3) presented odds ratio (OR), relative risk (RR), or hazard ratio (HR) estimates with 95% confidence intervals (CI) or data necessary to calculate them. Studies were excluded if they used a cross‐sectional study design. Studies primarily focusing on subjects with extensive exposure to radiation were not included because exposure to radiation is the most well‐established risk factor for thyroid cancer, and it would make the studied population significantly different from more general population and might induce bias for the research question of interest. Studies were included regardless of publication status, sample size and length of follow‐up. If multiple publications from the same study were identified, we included the study with the largest number of cases and most relevant information, like previous studies 24, 25, 26, 27.

### Data extraction and quality assessment

A pair of investigators independently carried out the abstract screening, full‐text screening, data extraction, and quality assessment. Disagreements were resolved by consensus. Data extracted from each study included: the first author's last name, year of publication, study region, study design, characteristics of study population (sample size, age, length of follow‐up, measures and numbers of parity, and effect sizes). If multiple estimates of the association for the same outcome were reported, we extracted the estimate that adjusted for the most appropriate covariates, like previous studies 28, 29. In cases when only unadjusted estimates were presented, we included the crude estimates. When the eligible studies did not present enough data, corresponding authors were contacted.

To assess the study quality, we used the Newcastle–Ottawa Quality Assessment Scale 30 in terms of population and sample methods, exposure and outcome descriptions, and statistical matching/adjustments of the data. This scale was used to assign a maximum of nine points for each study. Studies with score of seven or above were categorized as high‐quality studies, and those with score of 6 or below were categorized as low‐quality studies.

### Statistical methods

The RRs and corresponding 95% CIs from each of the included studies were used as the measure of association across studies. Due to the rarity of thyroid cancer, ORs and HRs were deemed equivalent to RRs and we used RRs to represent measures. We used the *I* ^2^ to assess the heterogeneity across the included studies, where *I* ^2^ > 50% suggests substantial heterogeneity 31. We pooled the log‐transformed RR using either the fixed‐effects model 32, 33 when there was no considerable heterogeneity or the random‐effects model 34 when there was substantial heterogeneity. Besides pooling results for parous versus nulliparous, we further conducted analyses summarizing effect sizes according to different number of parity. Based on the available data, we analyzed parity number of one versus nulliparous, parity number of two versus nulliparous, and parity number of three versus nulliparous, respectively. Subgroup analyses were conducted based on study design (case–control vs. prospective studies), geographic location (America, Europe, Asia, or Oceania), study quality (high vs. low), type of controls (population‐based vs. hospital‐based), and whether the study was adjusted for confounders (yes vs. no). We also conducted sensitivity analyses excluding one study at a time to explore whether any specific study strongly influenced the results.

For the dose–response analysis, we explored potential nonlinear and linear relationship between the number of parity and risk of thyroid cancer 35, 36. If studies reported the parity number by ranges, we set the midpoint of each category by averaging the lower and upper bound. If the highest category did not have an upper bound, we assumed that the open ended interval's width was as same as the adjacent interval's width. We examined a potential nonlinear dose–response relationship between parity and thyroid cancer with fractional polynomial models, using restricted cubic splines with three knots at fixed percentiles (10, 50, and 90%) of the distribution [37, 38]. We conducted a likelihood ratio test to evaluate the difference between the linear and nonlinear models^39^.

Publication bias was evaluated via Egger's test 40 and Begg's test 38. A *P*‐value of 0.05 was used as the threshold for determining significant publication bias. All statistical analyses were performed with Stata (version 13; StataCorp, College Station, TX).

## Results

### Literature search and study characteristics

**Figure 1:**
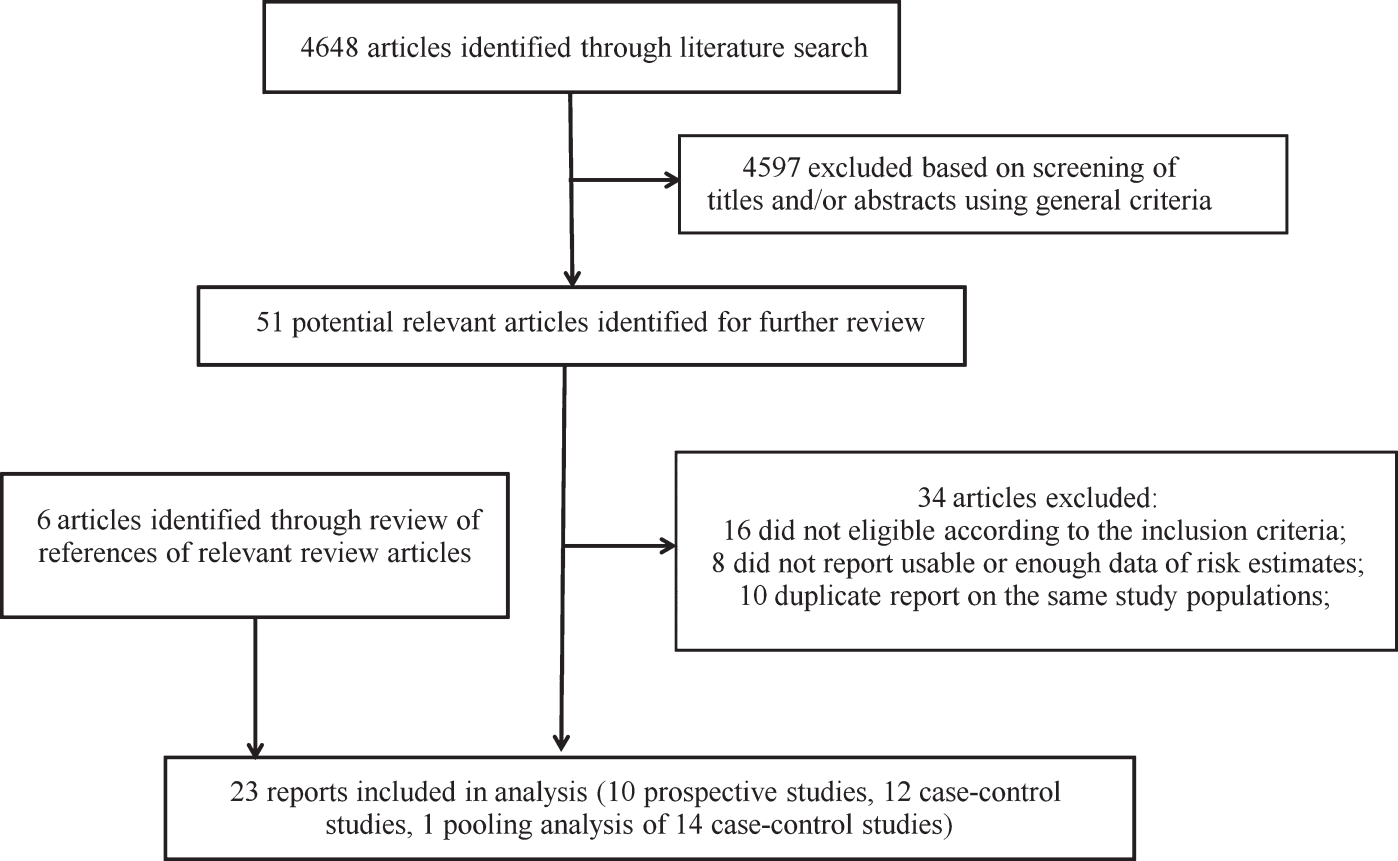
Flowchart for selection of eligible studies.

The detailed steps of the literature search were shown in Figure 1. After excluding 34 studies during the assessment of whole contents of 50 potential articles due to various reasons (the list of the 34 studies is available upon request), a total of 23 reports met the inclusion criteria and were included in this study 12, 15, 17, 18, 20, 21, 22, 39, 41, 42, 43, 44, 45, 46, 47, 48, 49, 50, 51, 52, 53, 54, 55. Since one study reported the risk estimates separately according to the age category (<45 years old or ≥45 years old) 12 and the combined effect size was unable to determine based on available data, we treated the two estimates as from two separate studies and incorporated both in the pooled analysis. The detailed characteristics of the included studies were shown in Table 1. In total, 10 prospective studies (seven cohort studies, one nested case–control study and two case cohort studies), 12 case–control studies and one pooling analysis of 14 case–control studies were available. Overall, eight studies were conducted in Europe, seven in America, five in Asia, two in Oceania, and one was conducted internationally. The studies enrolled 8860 patients and had a median follow‐up of 11 years (range 8.8–28 years). The detailed quality ratings for each study were listed in Tables 2 and 3. Overall, the studies had fair methodological quality. Fourteen studies had scores of seven or above and were categorized as high‐quality studies; eight studies were categorized as low‐quality studies. Parity was defined as full‐term pregnancies in 17 included studies 12, 15, 17, 18, 20, 21, 22, 39, 41, 43, 44, 47, 48, 49, 50, 53, 54, defined as pregnancies in three studies 42, 52, 55, defined as pregnancies lasting greater than 4 months in one study 45, and unspecified in two studies 46, 51. With regards to the histopathological types of thyroid cancer, in 18 studies, various subtypes of thyroid cancer were included 15, 17, 18, 20, 21, 22, 41, 42, 43, 44, 45, 46, 47, 48, 49, 52, 54, 55; in three studies, only papillary thyroid cancer was assessed 12, 39, 51; in one study, only sporadic medullary thyroid cancer was assessed 50; and in another study, it was unclear which subtypes of thyroid cancer were evaluated 53.

**Table 1:** Characteristics of studies of parity and thyroid cancer risk

First author, publication year, country, study design	Cases/subject (age), duration of follow‐up	Parity categories (exposure/case assessment)	RR (95% CI)	Matched/Adjusted factors
Case–control studies				
Xhaard (2014), France, PC‐CS	633/679 (10–40 years)	Nulliparous	1.0 (ref)	Ethnic group, level of education, height, BMI, smoking status, sex, age, region of residence
		1	0.9 (0.6–1.2)	
		2	1.1 (0.8–1.7)	
		≥3	1.5 (0.7–3.0)	
		(Trained interviewer/Cancer registry + pathology record)		
Truong (2005), New Caledonia, PC‐CS	293/354 (N/A)	Nulliparous	1.0 (ref)	Age, ethnic, gender, reference/diagnosis year
		Parous	1.2 (0.7–1.9)	
		1	1.0 (0.5–1.9)	
		2	0.7 (0.4–1.4)	
		3	1.4 (0.7–2.6)	
		4–5	1.1 (0.6–2.1)	
		6–7	1.6 (0.8–3.3)	
		≥8	2.2 (1.1–4.3)	
Zivaljevic (2003); Serbia, HC‐CS	204/204 (14–87 years)	Nulliparous	1.0 (ref)	Sex, age, place of residence, time of hospitalization
		1	0.65 (0.29–1.43)	
		2	1.12 (0.81–1.55)	
		≥3	1.16 (0.84–1.60)	
		(Trained interviewer/histological confirmed)	Individuals <45 years	
Sakoda (2002); USA, PC‐CS	608/558 (20–74 years)	Nulliparous	1.0 (ref)	Age, race/ethnic, history of radiation to the head or neck, history of goiter or nodules, family history of proliferative thyroid disease, education level, OC use, recency of last FTP, and birthplace
		Parous	1.4 (0.98–2.1)	
		1	1.2 (0.75–1.9)	
		2	1.7 (1.1–2.7)	
		≥3	1.4 (0.82–2.4)	
			Individuals ≥45 years	
		Nulliparous	1.0 (ref)	
		Parous	0.73 (0.42–1.3)	
		1	0.7 (0.34–1.5)	
		2	0.87 (0.47–1.6)	
		≥3	0.62 (0.34–1.2)	
		(Trained interviewer/Cancer registry)		
Memon (2002); Kuwait, PC‐CS	238/238 (10–65 years)	Nulliparous	1.0 (ref)	Age, gender, nationality, district of residence
		1–2	0.9 (0.5–1.8)	
		3–4	1.3 (0.6–2.5)	
		5–6	1.4 (0.7–2.8)	
		7–8	1.2 (0.6–2.6)	
		9–10	1.9 (0.8–4.9)	
		≥11	2.0 (0.7–5.8)	
		(Trained interviewer/medical record)		
Rossing (2000), Washington, USA, PC‐CS	410/574 (18–64 years)	Nulliparous	1.0 (ref)	Age, county of residence, race, marital status, cigarette smoking, alcohol consumption, history of radiation treatment to the head or neck as a child or adolescent, family history of thyroid cancer, use of oral contraceptives, history of benign thyroid disease
		1	0.9 (0.6–1.5)	
		2	0.9 (0.6–1.4)	
		3	1.2 (0.7–2.0)	
		≥4	1.1 (0.5–2.3)	
		(Trained interviewer/Cancer registry)		
Negri (1999), International, pooled analysis of case‐ control studies	2247/3699 (NA)	Nulliparous	1.0 (ref)	Study, age, history of radiation, oral contraceptive use
		Parous	1.2 (1.0–1.4)	
		1	1.3 (1.0–1.6)	
		2	1.2 (1.0–1.4)	
		3	1.1 (0.9–1.4)	
		≥4	1.2 (1.0–1.6)	
Brindel (2008), French Polynesia, PC‐CS	201/324 (NA)	Nulliparous	1.0 (ref)	Age
		Parous	1.7 (0.8–3.5)	
		1	0.9 (0.3–2.3)	
		2	1.6 (0.7–3.8)	
		3	2.3 (1.0–5.5)	
		4–5	2.2 (0.9–5.2)	
		6–7	2.7 (1.0–7.6)	
		≥8	1.7 (0.7–4.4)	
		(Trained interviewer/Cancer registry + pathology review)		
Kalezic (2013), Serbia, PC‐CS	98/196 (NA)	Nulliparous	1.0 (ref)	Age, place of residence
		Parous	0.7 (0.47–1.05)	
		(Trained interviewer/histopathological finding)		
Lee (2010), Korea, HC‐CS	260/259 (NA)	Nulliparous	1.0 (ref)	Age
		Parous	1.27 (0.88–1.84)	
		(Self‐questionnaire/unclear)		
Przybylik‐Mazurek (2012), Poland, HC‐CS	99/51 (mean 41/37)	Nulliparous	1.0 (ref)	Age, age of menarche, breastfeeding, estradiol, progesterone level
		Parous	1.52 (1.03–2.23)	
		1–2	3.03 (0.89–10.37)	
		≥3	6.16 (1.41–26.88)	
		(Self‐questionnaire/unclear)		
Takezaki (1996), Japan, HC‐CS	94/22666 (20–79)	Nulliparous	1.0 (ref)	Age, year of visit
		Parous	2.09 (1.05–4.15)	
		1–2	1.8 (0.9–3.7)	
		≥3	2.5 (1.1–5.7)	
		(Self‐questionnaire/histology confirmation)		
Lence‐Anta (2014), Cuba, PC‐CS	179/173 (17–60)	Nulliparous	1.0 (ref)	Age, smoking status, ethnic group, level of education, height, and BMI
		Parous	2.31 (1.22–4.39)	
		1	1.3 (0.5–3.4)	
		2	2.5 (1.1–6.1)	
		≥3	3.8 (1.7–8.3)	
		(Trained interviewer/Cancer registry + pathology register)		
Prospective studies				
Zamora‐Ros (2014), Europe, CS	508/345,157 (mean 51 years), 11 years	Nulliparous	1.0 (ref)	Age, study center, age at recruitment
		Parous	0.87 (0.66–1.15)	
		1	0.85 (0.61–1.20)	
		2	0.91 (0.66–1.22)	
		≥3	0.82 (0.59–1.12)	
		(Self‐questionnaire/Cancer registry)		
Kabat (2012), USA, CS	296/145,007 (50–79), 12.7 years	Nulliparous	1.0 (Ref)	Age, education, ethnicity, age at menarche, BMI, age at menopause, hormone therapy, physical activity, height, OC/CT, alcohol intake, pack‐years of smoking, and history of goiter/nodules, randomization status in each CT
		Parous	1.15 (0.72–1.85)	
		1–2	0.88 (0.53–1.47)	
		3–4	1.30 (0.89–1.89)	
		≥5	1.19 (0.77–1.84)	
		(Self‐questionnaire//Medical record and pathology report)		
Schonfeld (2011), USA, CS	312/187,865 (median 62.2), mean 9.3 years	Nulliparous	1.0 (Ref)	Unadjusted
		Parous	1.03 (0.74–1.45)	
		1–2	1.20 (0.84–1.71)	
		≥3	1.02 (0.72–1.45)	
		(Self‐questionnaire/Cancer registry)		
Pham (2009), Japan, CS	86/110,792 (40–79 years), 9 years	Nulliparous	1.0 (Ref)	Unadjusted
		1	0.45 (0.14–1.41)	
		2	0.59 (0.26–1.35)	
		3	0.55 (0.24–1.27)	
		≥4	0.32 (0.12–0.87)	
		(Self‐questionnaire/Cancer registry)		
Navarro Silvera (2005), Canada, CS	169/89,835 (40–59 years), 15.9 years	Nulliparous	1.0 (Ref)	Age, study center, randomization group, age at first live birth
		1–2	0.65 (0.35–1.23)	
		3–4	0.85 (0.47–1.54)	
		≥5	0.65 (0.32–1.33)	
		(Self‐questionnaire/cancer database)		
Galanti (1995), Sweden, NC‐CS	1409/7019 (15–59 years), 21 years	Nulliparous	1.0 (Ref)	Age
		1	1.2 (1.0–1.4)	
		2	1.1 (0.9–1.3)	
		3	1.2 (1.0–1.5)	
		≥4	1.1 (0.8–1.4)	
		(Registry/Cancer registry)		
Akslen (1992) Norway, CS	124/63,090 (32–74 years), 28 years	Nulliparous	1.0 (Ref)	Unadjusted
		Parous	0.97 (0.61–1.54)	
		1–2	0.98 (0.60–1.60)	
		≥3	0.99 (0.60–1.63)	
		(Trained interviewer/Cancer registry)		
Wong (2006), China, Case cohort study	130/3187 (30–69), 10 years	Nulliparous	1.0 (ref)	Age
		1	1.35 (0.20, 9.06)	
		≥2	0.32 (0.05, 2.15)	
		(Trained interviewer/Cancer registry)		
Hannibal (2008), Denmark, Case cohort study	29/54362 (median 30 years), median 8.8 years	Nulliparous	1.0 (ref)	Unadjusted
		Parous	0.75 (0.35–1.62)	
		1	0.83 (0.35–1.97)	
		≥2	0.68 (0.27–1.71)	
		(Trained interviewer/Cancer registry)		
Horn‐Ross (2011), USA, CS	233/117,646 (NA),~11 years	Nulliparous	1.0 (ref)	Unadjusted
		Parous	1.07 (0.80–1.44)	
		1–2	1.18 (0.86–1.60)	
		≥3	0.86 (0.59–1.26)	
		(Self‐questionnaire/Cancer registry)		

BMI: body mass index; CI: confidence interval; CS: cohort study; HC‐CS: hospital‐based case–control study; N/A: not available; NC‐CS: nested case–control study; OR: odds ratio; PC‐CS: population‐based case–control study; Ref: reference; RR: relative risk.

**Table 2:** Quality assessment of reviewed case–control studies

Study	Case defined with independent validation	Representativeness of the cases	Selection of controls from community	Statement that controls have no history of outcome	Cases and controls matched and/or adjusted by factors	Ascertain exposure by blinded structured interview	Same method of ascertainment for cases and controls	Same response rate for both groups
Sakoda (2002)	0	1	1	0	2	0	1	1
Memon (2002)	1	1	1	1	2	1	1	1
Rossing (2000)	1	1	1	0	2	1	1	1
Truong (2014)	1	1	1	1	2	1	1	1
Xhaard (2014)	1	1	1	1	2	1	1	1
Zivaljevic (2003)	1	1	0	0	2	1	1	1
Brindel (2008)	1	1	1	0	1	1	1	1
Kalezic (2013)	1	1	1	0	2	1	1	1
Lee (2010)	0	1	0	1	1	0	1	1
Przybylik‐Mazurek (2012)	0	0	0	1	2	0	1	1
Takezaki (1996)	1	1	0	1	2	0	1	1
Lence‐Anta (2014)	1	1	1	0	2	1	1	1

1 means study adequately fulfilled a quality criterion (2 for case–control fully matched and adjusted), 0 means it did not. Quality scale does not imply that items are of equal relevant importance.

**Table 3:** Quality assessment of reviewed prospective studies

Study	Exposed cohort represents average in community	Selection of the nonexposed cohort from same community	Ascertain exposure through records or structured interviews	Demonstrate that outcome not present at study start	Exposed and nonexposed matched and/or adjusted by factors	Ascertain outcome via independent blind assessment or record linkage	Follow‐up long enough for outcome to occur	Loss to follow‐up <20%
Akslen (1992)	1	1	1	0	0	1	1	1
Galanti (1995)	1	1	1	1	1	1	1	1
Kabat (2012)	1	1	0	0	2	1	1	1
Navarro Silvera (2005	1	1	0	0	2	1	1	1
Pham (2009)	1	1	0	1	0	1	1	1
Schonfeld (2011)	1	1	0	1	0	1	1	1
Zamora‐Ros (2014)	1	1	0	1	2	1	1	1
Wong (2006)	1	1	1	0	1	1	1	1
Hannibal (2008)	0	1	1	0	0	1	1	1
Horn‐Ross (2011)	1	1	0	1	0	1	1	1

1 means study adequately fulfilled a quality criterion, 0 means it did not. Quality scale does not imply that items are of equal relevant importance.

### Parous versus nulliparous

**Figure 2:**
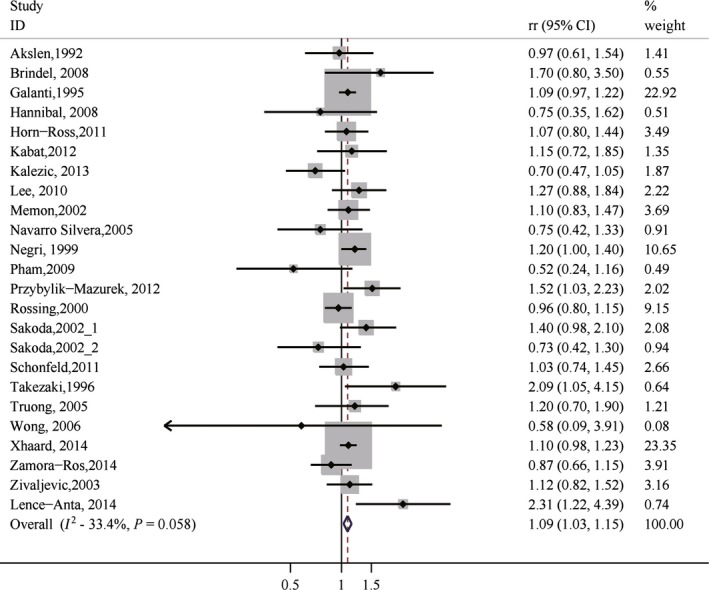
Forest plot (fixed‐effects model) of parity (parous vs. nulliparous) and thyroid cancer risk.

After pooling results from all available studies, there was a significant positive association between risk of thyroid cancer and parity for parous versus nulliparous (RR = 1.09, 95% CI 1.03–1.15), with no considerable heterogeneity (*I* ^2^ = 33.4%; Table 4 and Fig. 2). There was no significant publication bias as indicated by Egger's test (*P* for bias: 0.878) and Begg's test (*P* for bias: 1.000). Sensitivity analysis revealed that the 23 study‐specific RRs of parous versus nulliparous ranged from a low of 1.07 (95% CI 1.01–1.14; *I* ^2^ = 33.4%) after omission of the study by Negri et al. 22 to a high of 1.10 (95% CI 1.04–1.17; *I* ^2^ = 32.4%) after omission of the study by Rossing et al. 39. The subgroup analyses revealed that the significant positive association persisted in almost all strata, although the statistical significance was only achieved in some of them (Table 4).

**Table 4:** Summary risk estimates of the association between parity and thyroid cancer risk (parous vs. nulliparous)

	No of reports	RR (95% CI)	*I* ^2^	*P* for heterogeneity
Overall	24	**1.09 (1.03**–**1.15)**	33.4%	0.058
Subgroup analysis				
Study design				
Prospective	10	1.03 (0.94–1.13)	0.0%	0.558
Case–control	14	**1.12 (1.05**–**1.20)**	47.0%	0.027
Study quality				
High	14	**1.07 (1.00**–**1.14)**	38.4%	0.071
Low	9	1.11 (0.96–1.27)	31.8%	0.164
Location				
Europe	8	1.07 (0.996–1.15)	36.4%	0.139
America	8	1.04 (0.93–1.17)	41.7%	0.100
Asia	5	1.15 (0.94–1.41)	47.9%	0.104
Oceania	2	1.34 (0.89–2.02)	0.0%	0.444
International	1	**1.20 (1.00**–**1.40)**	–	–
Type of controls				
Population‐based	9	1.07 (0.99–1.17)	52.5%	0.032
Hospital‐based	4	**1.32 (1.08**–**1.60)**	10.7%	0.339
Confounder adjustment				
Yes	19	**1.10 (1.04**–**1.16)**	39.6%	0.039
No	5	0.98 (0.81–1.18)	0.0%	0.496

significant associations are bolded.

### Different number of parity

We assessed the associations between different number of parity (1, 2 or 3) and risk of thyroid cancer, respectively (Table 5). Parity number of one versus nulliparous was positively associated with risk of thyroid cancer (RR = 1.08, 95% CI 0.98–1.21; *I* ^2^ = 3.6%), although the association was not statistically significant. On the other hand, both parity number of two versus nulliparous and parity number of three versus nulliparous demonstrated significant positive association with the risk of thyroid cancer (RR = 1.11, 95% CI 1.01–1.22; *I* ^2^ = 31.1% and RR = 1.16, 95% CI 1.01–1.33; *I* ^2^ = 19.6%, respectively).

**Table 5:** Summary risk estimates of the associations between different number of parity and thyroid cancer risk

	No of reports	RR (95% CI)	*I* ^2^	*P* for heterogeneity
Parity number of one versus nulliparous	14	1.08 (0.98–1.21)	3.6%	0.411
Parity number of two versus nulliparous	12	1.11 (1.01–1.22)	31.1%	0.142
Parity number of three versus nulliparous	6	1.16 (1.01–1.33)	19.6%	0.285

### Dose–response meta‐analysis

Based on the dose–response analysis, we did not detect a nonlinear dose–response relationship between the number of parity and risk of thyroid cancer. Assuming a linear dose–response relationship, the combined RR per live birth was 1.01 (95% CI 0.96–1.07; *P* = 0.69 for the linear trend), with significant heterogeneity (*P* for heterogeneity: <0.0001). There seemed not be a clear dose–response relationship between the number of parity and thyroid cancer risk.

## Discussion

### Main findings

We performed a comprehensive systematic review and meta‐analysis to assess the association between parity and risk of thyroid cancer. After summarizing available evidence from observational studies, ever giving birth to children was identified to be significantly associated with an increased risk of developing thyroid cancer. Analyses assessing different numbers of parity (1, 2 and 3) demonstrated that such a significant positive association with thyroid cancer risk persisted for both parity number of two versus nulliparous and parity number of three versus nulliparous. However, the dose–response analysis did not suggest a significant nonlinear or linear relationship between the number of parity and thyroid cancer risk. Overall, these findings suggested that parity might be associated with risk of thyroid cancer in females, while the exact relationship needs exploration and clarification in further studies.

### Interpretation

Although the exact biological mechanism underlying the potential association between parity and risk of thyroid cancer has not been completely established, plausible explanations have been suggested by basic research. During pregnancy estrogens are elevated, which potentially influence the proliferation as well as enhance the adhesion, migration, and invasiveness of malignant thyroid cells 56, 57, 58. Estrogens are also known to interact with estrogen receptors and alter apoptotic pathways, which are suggested to be linked to tumor development 59, 60, 61.

In the subgroup, analyses of the association between thyroid cancer risk and parity for parous versus nulliparous, significant positive association was also detected in subgroups of studies with a case–control design, case–control studies with hospital‐based controls, high–quality studies, and studies with confounder adjustments. We acknowledge that studies with a case–control design are more susceptible to bias compared with studies with a prospective design. Similarly, case–control studies with hospital‐based controls may be more susceptible to bias compared with those with population‐based controls. On the other hand, even though the detected associations in many other subgroups did not reach statistical significance, the directions of the associations tend to be positive. The trend of a positive association was also suggested for parity number of one versus nulliparous. These suggest that the detected positive association between parity and thyroid cancer risk may be real and warrants further clarification.

Several reasons may explain the inconsistencies of the association between parity and thyroid cancer across included studies. For example, not all included studies sufficiently adjust for relevant covariates. Besides parity, several other reproductive factors like age at first pregnancy, OC use, and age at menopause are suggested to influence thyroid cancer risk as well 13, 43, 62. These relevant factors may vary across different countries where the included studies were conducted. This may partially explain some of the inconsistencies of the association of interest.

### Strengths and limitations

Our study has several strengths. To the best of our knowledge, this is the most comprehensive meta‐analysis evaluating the association between parity and thyroid cancer risk. A systematic review previously assessed the association 6; however, instead of quantitatively evaluating the evidence, they just briefly discussed the risk estimate trends. After the conduction of this study, several meta‐analysis studies evaluating a similar research question were published 63, 64. We think this study has advantages compared with those studies: for the study by Zhou et al. 64, the literature was only updated through April 2013, and a couple of more recent studies were not included in their analysis 43, 47, 50; for the study by Caini et al. 63, evidence from case–control studies were not included. Furthermore, ours is the first study assessing the dose–response relationship to better characterize the relationship. Our study quantitatively summarized all available evidence from epidemiological studies and might have sufficient power to assess the association of interest. Besides conducting subgroup analyses and sensitivity analyses, we also assessed associations according to different numbers of parity and conducted dose–response analysis with the aim of fully understanding the relationship.

Several potential limitations must be acknowledged for the interpretation of our findings. First, we did not have access to the individualized primary data from the included studies, and the risk estimates used in pooling might not be fully adjusted for. Relevant covariates including age, BMI, iodine intake, use of OC, HRT, and fertility treatment were not always adjusted for in the included studies. Residual confounding may thus be an issue for our findings. Further well‐designed studies with full adjustments are needed. Second, during the dose–response analysis, the highest levels of number of parity in different studies have wide range of values, which may cause the exposure values to not be accurately assigned. This may be one reason that we did not detect a linear or nonlinear dose–response relationship between the number of parity and risk of thyroid cancer, which seemed to be suggested based on the increasing risks over parity of 1, 2, and 3 (Table 2). However, this is a known shortcoming for determining the dose–response relationship with aggregate data. The dose–response relationship of parity and thyroid cancer risk is thus warranted to be further explored in well‐designed studies.

## Conclusion

Based on a summarization of relevant evidence from epidemiological studies, parous versus nulliparous was positively associated with risk of thyroid cancer. A similar positive association was also detected for both parity number of two versus nulliparous and parity number of three versus nulliparous. However, no linear or nonlinear relationship between the number of parity and thyroid cancer risk was detected. Although parity might be associated with the risk of thyroid cancer in females, further studies are warranted to better clarify the relationship.

## Conflict of Interest

There are no competing interests to declare.

## References

[1] Wartofsky, L. 2010 Increasing world incidence of thyroid cancer: increased detection or higher radiation exposure? Hormones (Athens). 9:103–108. 20687393 10.14310/horm.2002.1260

[2] Siegel, R. L. , K. D. Miller , and A. Jemal . 2015 Cancer statistics, 2015. CA Cancer J. Clin. 65:5–29. doi: 10.3322/caac.21254. 25559415 10.3322/caac.21254

[3] Sakorafas, G. H. , H. Friess , and G. Peros . 2008 The genetic basis of hereditary medullary thyroid cancer: clinical implications for the surgeon, with a particular emphasis on the role of prophylactic thyroidectomy. Endocr. Relat. Cancer 15:871–884. doi: 10.1677/ERC‐08‐0098. 19015274 10.1677/ERC-08-0098

[4] Musholt, T. J. , P. B. Musholt , T. Petrich , G. Oetting , W. H. Knapp , and J. Klempnauer . 2000 Familial papillary thyroid carcinoma: genetics, criteria for diagnosis, clinical features, and surgical treatment. World J. Surg. 24:1409–1417. 11038215 10.1007/s002680010233

[5] Pacini, F. , M. G. Castagna , L. Brilli , G. Pentheroudakis , and E. G. W. Group . 2012 Thyroid cancer: ESMO Clinical Practice Guidelines for diagnosis, treatment and follow‐up. Ann. Oncol. 23(Suppl 7):vii110–vii119. doi:10.1093/annonc/mds230. 22997443 10.1093/annonc/mds230

[6] Peterson, E. , P. De , and R. Nuttall . 2012 BMI, diet and female reproductive factors as risks for thyroid cancer: a systematic review. PLoS ONE 7:e29177. doi: 10.1371/journal.pone.0029177. 22276106 10.1371/journal.pone.0029177 PMC3261873

[7] Dal Maso, L. , C. Bosetti , C. La Vecchia , and S. Franceschi . 2009 Risk factors for thyroid cancer: an epidemiological review focused on nutritional factors. Cancer Causes Control 20:75–86. 18766448 10.1007/s10552-008-9219-5

[8] Jing, Z. , X. Hou , Y. Liu , S. Yan , R. Wang , S. Zhao , et al. 2015 Association between height and thyroid cancer risk: a meta‐analysis of prospective cohort studies. Int. J. Cancer 137:1484–1490. doi: 10.1002/ijc.29487. 25693727 10.1002/ijc.29487

[9] Yeo, Y. , S. H. Ma , Y. Hwang , P. L. Horn‐Ross , A. Hsing , K. E. Lee , et al. 2014 Diabetes mellitus and risk of thyroid cancer: a meta‐analysis. PLoS ONE 9:e98135. doi: 10.1371/journal.pone.0098135. 24927125 10.1371/journal.pone.0098135 PMC4057085

[10] Zhao, Z. G. , X. G. Guo , C. X. Ba , W. Wang , Y. Y. Yang , J. Wang , et al. 2012 Overweight, obesity and thyroid cancer risk: a meta‐analysis of cohort studies. J. Int. Med. Res. 40:2041–2050. 23321160 10.1177/030006051204000601

[11] Kitahara, C. M. , M. S. Linet , L. E. Beane Freeman , D. P. Check , T. R. Church , Y. Park , et al. 2012 Cigarette smoking, alcohol intake, and thyroid cancer risk: a pooled analysis of five prospective studies in the United States. Cancer Causes Control 23:1615–1624. doi: 10.1007/s10552‐012‐0039‐2. 22843022 10.1007/s10552-012-0039-2 PMC3511822

[12] Sakoda, L. C. , and P. L. Horn‐Ross . 2002 Reproductive and menstrual history and papillary thyroid cancer risk: the San Francisco Bay Area thyroid cancer study. Cancer Epidemiol. Biomark. Prev. 11:51–57. 11815401

[13] Wu, L. , and J. Zhu . 2015 Linear reduction in thyroid cancer risk by oral contraceptive use: a dose‐response meta‐analysis of prospective cohort studies. Hum. Reprod. 30:2234–2240. doi: 10.1093/humrep/dev160. 26141711 10.1093/humrep/dev160

[14] McTiernan, A. M. , N. S. Weiss , and J. R. Daling . 1984 Incidence of thyroid cancer in women in relation to reproductive and hormonal factors. Am. J. Epidemiol. 120:423–435. 6475918 10.1093/oxfordjournals.aje.a113907

[15] Truong, T. , L. Orsi , D. Dubourdieu , Y. Rougier , D. Hemon , and P. Guenel . 2005 Role of goiter and of menstrual and reproductive factors in thyroid cancer: a population‐based case‐control study in New Caledonia (South Pacific), a very high incidence area. Am. J. Epidemiol. 161:1056–1065. doi: 10.1093/aje/kwi136. 15901626 10.1093/aje/kwi136 PMC2668936

[16] Braganza, M. Z. , A. B. de Gonzalez , S. J. Schonfeld , N. Wentzensen , A. V. Brenner , and C. M. Kitahara . 2014 Benign breast and gynecologic conditions, reproductive and hormonal factors, and risk of thyroid cancer. Cancer Prev. Res. (Phila.) 7:418–425. doi: 10.1158/1940‐6207.CAPR‐13‐0367. 24449056 10.1158/1940-6207.CAPR-13-0367 PMC3976437

[17] Pham, T. M. , Y. Fujino , H. Mikami , N. Okamoto , Y. Hoshiyama , A. Tamakoshi , et al. 2009 Reproductive and menstrual factors and thyroid cancer among Japanese women: the Japan Collaborative Cohort Study. J Womens Health (Larchmt). 18:331–335. doi: 10.1089/jwh.2008.1038. 19281316 10.1089/jwh.2008.1038

[18] Wong, E. Y. , R. Ray , D. L. Gao , K. J. Wernli , W. Li , E. D. Fitzgibbons , et al. 2006 Reproductive history, occupational exposures, and thyroid cancer risk among women textile workers in Shanghai. China. Int Arch Occup Environ Health. 79:251–258. doi: 10.1007/s00420‐005‐0036‐9. 16220287 10.1007/s00420-005-0036-9

[19] Preston‐Martin, S. , F. Jin , M. J. Duda , and W. J. Mack . 1993 A case‐control study of thyroid cancer in women under age 55 in Shanghai (People's Republic of China). Cancer Causes Control 4:431–440. 8218875 10.1007/BF00050862

[20] Galanti, M. R. , M. Lambe , A. Ekbom , P. Sparen , and B. Pettersson . 1995 Parity and risk of thyroid cancer: a nested case‐control study of a nationwide Swedish cohort. Cancer Causes Control 6:37–44. 7718734 10.1007/BF00051679

[21] Schonfeld, S. J. , E. Ron , C. M. Kitahara , A. Brenner , Y. Park , A. J. Sigurdson , et al. 2011 Hormonal and reproductive factors and risk of postmenopausal thyroid cancer in the NIH‐AARP Diet and Health Study. Cancer Epidemiol. 35:e85–e90. doi: 10.1016/j.canep.2011.05.009. 21852218 10.1016/j.canep.2011.05.009 PMC3215902

[22] Negri, E. , L. Dal Maso , E. Ron , C. La Vecchia , S. D. Mark , S. Preston‐Martin , et al. 1999 A pooled analysis of case‐control studies of thyroid cancer. II. Menstrual and reproductive factors. Cancer Causes Control 10:143–155. 10231163 10.1023/a:1008880429862

[23] Stroup, D. F. , J. A. Berlin , S. C. Morton , I. Olkin , G. D. Williamson , D. Rennie , et al. 2000 Meta‐analysis of observational studies in epidemiology: a proposal for reporting. Meta‐analysis Of Observational Studies in Epidemiology (MOOSE) group. JAMA 283:2008–2012. 10789670 10.1001/jama.283.15.2008

[24] Wu, L. , Z. Wang , J. Zhu , A. Murad , L. Prokop , and M. Murad . 2015 Nut consumption and risk of cancer and type 2 diabetes: a systematic review and meta‐analysis. Nutr. Rev. 73:409–425. doi: 10.1093/nutrit/nuv006. 26081452 10.1093/nutrit/nuv006 PMC4560032

[25] Wu, L. , J. Zhu , L. Prokop , and M. Murad . 2015 Pharmacologic therapy of diabetes and overall cancer risk and mortality: a meta‐analysis of 265 studies. Sci. Rep. 5:10147. 26076034 10.1038/srep10147 PMC4467243

[26] Wu, Q. J. , L. Wu , L. Q. Zheng , X. Xu , C. Ji , and T. T. Gong . 2015 Consumption of fruit and vegetables reduces risk of pancreatic cancer: evidence from epidemiological studies. Eur. J. Cancer Prev. Epub 2015 Jun 11. doi: 10.1097/CEJ.0000000000000171. 10.1097/CEJ.0000000000000171 26075658

[27] Wang, Y. Z. , Q. J. Wu , J. Zhu , and L. Wu . 2015 Fish consumption and risk of myeloma: a meta‐analysis of epidemiological studies. Cancer Causes Control 26:1307–1314. doi: 10.1007/s10552‐015‐0625‐1. 26156047 10.1007/s10552-015-0625-1

[28] Guan, H. B. , L. Wu , Q. J. Wu , J. Zhu , and T. Gong . 2014 Parity and pancreatic cancer risk: a dose‐response meta‐analysis of epidemiologic studies. PLoS ONE 9:e92738. doi: 10.1371/journal.pone.0092738. 24658609 10.1371/journal.pone.0092738 PMC3962437

[29] Wu, Q. J. , C. Tu , Y. Y. Li , J. Zhu , K. Q. Qian , W. J. Li , et al. 2015 Statin use and breast cancer survival and risk: a systematic review and meta‐analysis. Oncotarget. Epub 2015 Oct 12. doi: 10.18632/oncotarget.5557. 10.18632/oncotarget.5557 PMC4767486 26472026

[30] Wells, G. A. , B. Shea , D. O'Connell , J. Peterson , V. Welch , M. Losos , et al. The Newcastle‐Ottawa Scale (NOS) for assessing the quality of nonrandomised studies in meta‐analyses. Available at http://www.ohri.ca/programs/clinical_epidemiology/oxford.asp (accessed May 15 2015).

[31] Higgins, J. P. , S. G. Thompson , J. J. Deeks , and D. G. Altman . 2003 Measuring inconsistency in meta‐analyses. BMJ 327:557–560. doi: 10.1136/bmj.327.7414.557. 12958120 10.1136/bmj.327.7414.557 PMC192859

[32] Higgins, J. P. , and S. G. Thompson . 2002 Quantifying heterogeneity in a meta‐analysis. Stat. Med. 21:1539–1558. doi: 10.1002/sim.1186. 12111919 10.1002/sim.1186

[33] Wu, Q. J. , Y. Y. Li , C. Tu , J. Zhu , K. Q. Qian , T. B. Feng , et al. 2015 Parity and endometrial cancer risk: a meta‐analysis of epidemiological studies. Sci. Rep. 5:14243. doi: 10.1038/srep14243. 26373341 10.1038/srep14243 PMC4642705

[34] DerSimonian, R. , and N. Laird . 1986 Meta‐analysis in clinical trials. Control. Clin. Trials 7:177–188. 3802833 10.1016/0197-2456(86)90046-2

[35] Greenland, S. , and M. P. Longnecker . 1992 Methods for trend estimation from summarized dose‐response data, with applications to meta‐analysis. Am. J. Epidemiol. 135:1301–1309. 1626547 10.1093/oxfordjournals.aje.a116237

[36] Orsini, N. , R. Li , A. Wolk , P. Khudyakov , and D. Spiegelman . 2012 Meta‐analysis for linear and nonlinear dose‐response relations: examples, an evaluation of approximations, and software. Am. J. Epidemiol. 175:66–73. doi: 10.1093/aje/kwr265. 22135359 10.1093/aje/kwr265 PMC3244608

[37] Liu, Q. , N. R. Cook , A. Bergstrom , and C. C. Hsieh . 2009 A two‐stage hierarchical regression model for meta‐analysis of epidemiologic nonlinear dose–response data. Comput. Stat. Data Anal. 53:4157–4167.

[38] Begg, C. B. , and M. Mazumdar . 1994 Operating characteristics of a rank correlation test for publication bias. Biometrics 50:1088–1101. 7786990

[39] Rossing, M. A. , L. F. Voigt , K. G. Wicklund , and J. R. Daling . 2000 Reproductive factors and risk of papillary thyroid cancer in women. Am. J. Epidemiol. 151:765–772. 10965973 10.1093/oxfordjournals.aje.a010276

[40] Egger, M. , G. Davey Smith , M. Schneider , and C. Minder . 1997 Bias in meta‐analysis detected by a simple, graphical test. BMJ 315:629–634. 9310563 10.1136/bmj.315.7109.629 PMC2127453

[41] Memon, A. , M. Darif , K. Al‐Saleh , and A. Suresh . 2002 Epidemiology of reproductive and hormonal factors in thyroid cancer: evidence from a case‐control study in the Middle East. Int. J. Cancer 97:82–89. 11774247 10.1002/ijc.1573

[42] Zivaljevic, V. , H. Vlajinac , R. Jankovic , J. Marinkovic , R. Dzodic , S. Sipeti Grujii , et al. 2003 Case‐control study of female thyroid cancer–menstrual, reproductive and hormonal factors. Eur. J. Cancer Prev. 12:63–66. doi: 10.1097/01.cej.0000051107.66188.86. 12548112 10.1097/00008469-200302000-00010

[43] Xhaard, C. , C. Rubino , E. Clero , S. Maillard , Y. Ren , F. Borson‐Chazot , et al. 2014 Menstrual and reproductive factors in the risk of differentiated thyroid carcinoma in young women in france: a population‐based case‐control study. Am. J. Epidemiol. 180:1007–1017. doi: 10.1093/aje/kwu220. 25269571 10.1093/aje/kwu220

[44] Akslen, L. A. , S. Nilssen , and G. Kvale . 1992 Reproductive factors and risk of thyroid cancer. A prospective study of 63,090 women from Norway. Br. J. Cancer 65:772–774. 1586606 10.1038/bjc.1992.163 PMC1977387

[45] Navarro Silvera, S. A. , A. B. Miller , and T. E. Rohan . 2005 Risk factors for thyroid cancer: a prospective cohort study. Int. J. Cancer 116:433–438. doi: 10.1002/ijc.21079. 15818623 10.1002/ijc.21079

[46] Kabat, G. C. , M. Y. Kim , J. Wactawski‐Wende , D. Lane , S. Wassertheil‐Smoller , and T. E. Rohan . 2012 Menstrual and reproductive factors, exogenous hormone use, and risk of thyroid carcinoma in postmenopausal women. Cancer Causes Control 23:2031–2040. doi: 10.1007/s10552‐012‐0084‐x. 23090034 10.1007/s10552-012-0084-x

[47] Zamora‐Ros, R. , S. Rinaldi , C. Biessy , A. Tjonneland , J. Halkjaer , A. Fournier , et al. 2015 Reproductive and menstrual factors and risk of differentiated thyroid carcinoma: the EPIC study. Int. J. Cancer 5:1218–1227. doi: 10.1002/ijc.29067. 25041790 10.1002/ijc.29067

[48] Brindel, P. , F. Doyon , F. Rachedi , J. L. Boissin , J. Sebbag , L. Shan , et al. 2008 Menstrual and reproductive factors in the risk of differentiated thyroid carcinoma in native women in French Polynesia: a population‐based case‐control study. Am. J. Epidemiol. 167:219–229. doi: 10.1093/aje/kwm288. 17965111 10.1093/aje/kwm288

[49] Hannibal, C. G. , A. Jensen , H. Sharif , and S. K. Kjaer . 2008 Risk of thyroid cancer after exposure to fertility drugs: results from a large Danish cohort study. Hum. Reprod. 23:451–456. doi: 10.1093/humrep/dem381. 18065402 10.1093/humrep/dem381

[50] Kalezic, N. K. , V. R. Zivaljevic , N. A. Slijepcevic , I. R. Paunovic , A. D. Diklic , and S. B. Sipetic . 2013 Risk factors for sporadic medullary thyroid carcinoma. Eur. J. Cancer Prev. 22:262–267. doi: 10.1097/CEJ.0b013e3283592c78. 22960778 10.1097/CEJ.0b013e3283592c78

[51] Horn‐Ross, P. L. , A. J. Canchola , H. Ma , P. Reynolds , and L. Bernstein . 2011 Hormonal factors and the risk of papillary thyroid cancer in the California Teachers Study cohort. Cancer Epidemiol. Biomark. Prev. 20:1751–1759. doi: 10.1158/1055‐9965.EPI‐11‐0381. 10.1158/1055-9965.EPI-11-0381 PMC3288117 21791618

[52] Przybylik‐Mazurek, E. , A. Hubalewska‐Dydejczyk , A. Fedorowicz , and D. Pach . 2012 Factors connected with the female sex seem to play an important role in differentiated thyroid cancer. Gynecol. Endocrinol. 28:150–155. doi: 10.3109/09513590.2011.563909. 21663528 10.3109/09513590.2011.563909

[53] Lee, S. M. , and K. H. Kwak . 2010 Risk factors and a predictive model for thyroid cancer in Korean women. Cancer Nurs. 33:310–319. doi: 10.1097/NCC.0b013e3181cd2844. 20495449 10.1097/NCC.0b013e3181cd2844

[54] Takezaki, T. , K. Hirose , M. Inoue , N. Hamajima , T. Kuroishi , S. Nakamura , et al. 1996 Risk factors of thyroid cancer among women in Tokai, Japan. J. Epidemiol. 6:140–147. 8952218 10.2188/jea.6.140

[55] Lence‐Anta, J. J. , C. Xhaard , R. M. Ortiz , H. Kassim , C. M. Pereda , S. Turcios , et al. 2014 Environmental, lifestyle, and anthropometric risk factors for differentiated thyroid cancer in cuba: a case‐control study. Eur Thyroid J. 3:189–196. doi: 10.1159/000362928. 25538901 10.1159/000362928 PMC4224259

[56] Manole, D. , B. Schildknecht , B. Gosnell , E. Adams , and M. Derwahl . 2001 Estrogen promotes growth of human thyroid tumor cells by different molecular mechanisms. J. Clin. Endocrinol. Metab. 86:1072–1077. doi: 10.1210/jcem.86.3.7283. 11238488 10.1210/jcem.86.3.7283

[57] Glinoer, D. , P. de Nayer , P. Bourdoux , M. Lemone , C. Robyn , A. van Steirteghem , et al. 1990 Regulation of maternal thyroid during pregnancy. J. Clin. Endocrinol. Metab. 71:276–287. doi: 10.1210/jcem‐71‐2‐276. 2116437 10.1210/jcem-71-2-276

[58] Rajoria, S. , R. Suriano , A. Shanmugam , Y. L. Wilson , S. P. Schantz , J. Geliebter , et al. 2010 Metastatic phenotype is regulated by estrogen in thyroid cells. Thyroid 20:33–41. doi: 10.1089/thy.2009.0296. 20067378 10.1089/thy.2009.0296 PMC2833180

[59] Lee, M. L. , G. G. Chen , A. C. Vlantis , G. M. Tse , B. C. Leung , and C. A. van Hasselt . 2005 Induction of thyroid papillary carcinoma cell proliferation by estrogen is associated with an altered expression of Bcl‐xL. Cancer J. 11:113–121. 15969986 10.1097/00130404-200503000-00006

[60] Bouman, A. , M. J. Heineman , and M. M. Faas . 2005 Sex hormones and the immune response in humans. Hum Reprod Update. 11:411–423. doi: 10.1093/humupd/dmi008. 15817524 10.1093/humupd/dmi008

[61] Chen, G. G. , A. C. Vlantis , Q. Zeng , and C. A. van Hasselt . 2008 Regulation of cell growth by estrogen signaling and potential targets in thyroid cancer. Curr. Cancer Drug Targets 8:367–377. 18690843 10.2174/156800908785133150

[62] Cao, Y. W. Z. , J. Gu , F. Hu , Y. Qi , Q. Yin , et al. 2015 Reproductive factors but not hormonal factors associated with thyroid cancer risk: a systematic review and meta‐analysis. BioMed Research International. 2015:103515. doi: 10.1155/2015/103515. 10.1155/2015/103515 PMC4538312 26339585

[63] Caini, S. , B. Gibelli , D. Palli , C. Saieva , M. Ruscica , and S. Gandini . 2015 Menstrual and reproductive history and use of exogenous sex hormones and risk of thyroid cancer among women: a meta‐analysis of prospective studies. Cancer Causes Control 26:511–518. doi: 10.1007/s10552‐015‐0546‐z. 25754110 10.1007/s10552-015-0546-z

[64] Zhou, Y. Q. , Z. Zhou , M. F. Qian , T. Gong , and J. D. Wang . 2015 Association of thyroid carcinoma with pregnancy: a meta‐analysis. Mol Clin Oncol. 3:341–346. doi: 10.3892/mco.2014.472. 25798264 10.3892/mco.2014.472 PMC4360860

